# Cu(OTf)_2_-catalyzed multicomponent reactions

**DOI:** 10.3762/bjoc.21.7

**Published:** 2025-01-14

**Authors:** Sara Colombo, Camilla Loro, Egle M Beccalli, Gianluigi Broggini, Marta Papis

**Affiliations:** 1 Dipartimento di Scienza e Alta Tecnologia, Università degli Studi dell’Insubria, Via Valleggio 9, 22100, Como, Italy; 2 DISFARM, Sezione di Chimica Generale e Organica “A. Marchesini”, Università degli Studi di Milano, Via Venezian 21, 20133, Milano, Italy

**Keywords:** cascade process, copper catalysis, heteropolycycles, multicomponent reactions, one-pot reaction

## Abstract

This review reports the achievements in copper(II) triflate-catalyzed processes concerning the multicomponent reactions, applied to the synthesis of acyclic and cyclic compounds. In particular, for the heteropolycyclic systems mechanistic insights were outlined as well as cycloaddition and aza-Diels–Alder reactions were included. These strategies have gained attention due to their highly atom- and step-economy, one-step multi-bond forming, mild reaction conditions, low cost and easy handling.

## Introduction

Copper has gained a relevant role in organic synthesis as an alternative to precious metals due to its low toxicity, ease of handling, high catalytic activity, and cost-effectiveness [1–2]. In recent years, Cu(OTf)_2_ has significantly emerged among copper catalysts because it can act as a precursor to triflic acid in addition to a powerful copper-catalytic effect. Indeed, Cu(OTf)_2_ has proven to be an excellent surrogate for triflic acid compared with other metal triflates because it is inexpensive and exhibits high activity with low toxicity [3–7].

Multicomponent reactions are one of the most effective methods to assemble multiple reagents, thus facilitating access to the target molecules more quickly, due to atom/step economy, short reaction times, and eco-friendly benefits. Combining multicomponent reactions with transition-metal catalysts provides synthetic tools even more advantageously. Copper has also become very interesting in this field, mainly in processes aimed at synthesizing heterocyclic compounds. Among the various catalysts, Cu(OTf)_2_ stands out in heterocyclic synthesis and ring transformations due to its dual activity as a metal catalyst as well as a Lewis acid [8–11]. However, in many cases, the role of copper is not clear and both activities often work synergistically. In all other cases, copper’s activity is due to the coordination/complexation with unsaturated systems, but it is rarely possible to exclude its action also as Lewis acid. Confirming this dual activity, it should be noted that copper triflate can rarely be replaced by other copper salts or complexes to obtain the same results. In general, catalyst switching does not work with copper triflate, thus supporting its unique behavior or reactivity properties. The ambiguity related to the role of Cu(OTf)_2_ is particularly relevant for cycloaddition reactions, where it is even more difficult to justify the activation of the copper species as a Lewis acid or metal catalyst [12–14].

**Figure 1:**
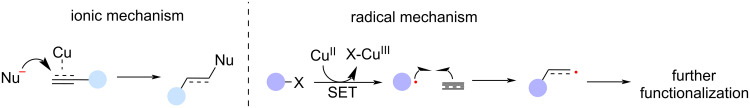
Plausible general catalytic activation for ionic or radical mechanisms.

The reaction mechanism involved can be ionic or radical (Figure 1). The latter is typically operative when the reaction is carried out under oxidative conditions, usually in the presence of O_2_ and TEMPO, involving the formation of radical species through single-electron transfer (SET) from a copper catalyst to a precursor. Subsequent addition to multiple C–C bonds generates extended carbon radicals capable of giving further functionalization.

Regarding the ionic mechanism, the key step generally comprises the complexation with the unsaturated substrate leading to activation of the alkenyl/alkynyl moiety towards a nucleophilic attack. In some cases, activation of a carbonyl group by the copper catalyst to facilitate nucleophilic attack has also been reported. Moreover, both activations can be operative simultaneously. Since copper shows affinity either for multiple C–C bonds or polar functional groups, it seems the ideal tool for this type of reaction.

## Review

### Three-component reactions

Several three-component procedures have been successfully carried out using Cu(OTf)_2_ as a catalyst. These processes have been exploited mainly to access nitrogen compounds endowed with various structures, in a faster and more sustainable way than reactions conducted step by step.

#### Providing acyclic compounds

**Scheme 1:**
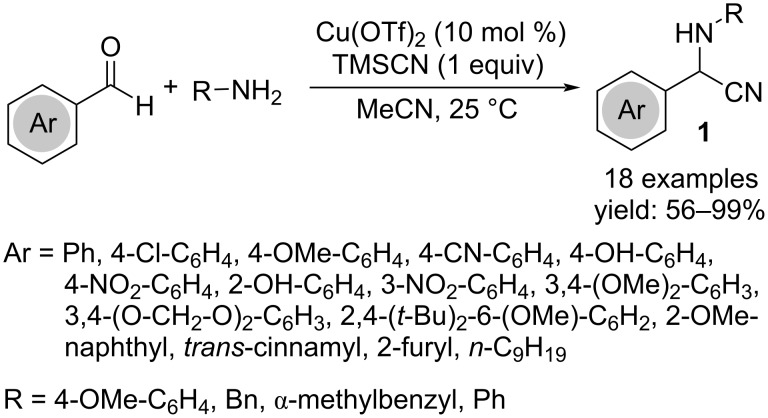
Synthesis of α-aminonitriles **1**.

A three-component Strecker-type condensation of aromatic aldehydes, amines, and cyanides under mild reaction conditions furnishes α-aminonitriles **1** in good to high yields (Scheme 1) [15]. The reaction failed only in the case of acetophenone. Among various Lewis acids, only Cu(OTf)_2_ in combination with TMSCN was effective or a valuable alternative was the use of acetone cyanohydrin combined with a catalytic amount of TEA (5 mol %). The mechanism involves the formation of an imine facilitating the addition of the nitrile group.

**Scheme 2:**
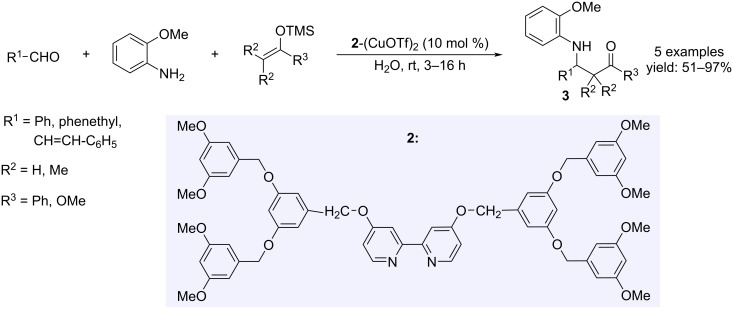
Synthesis of β-amino ketone or β-amino ester derivatives **3**.

Among the known processes, a particular Mannich-type reaction was realized in water in the presence of a dendritic 2,2’-bipyridine ligand **2** and Cu(OTf)_2_ (Scheme 2) [16]. The hydrophobic ligand surrounding the metal revealed to be essential for the organic synthesis in water, thus increasing the reaction yields.

**Scheme 3:**
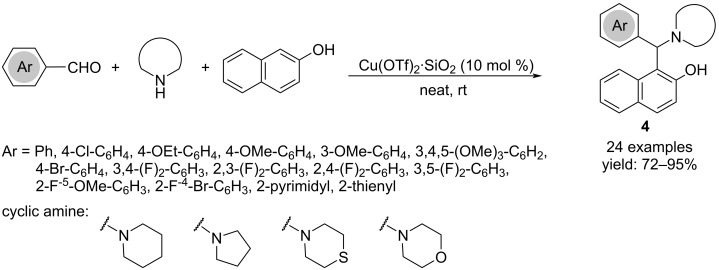
Synthesis of 1-(α-aminoalkyl)-2-naphthol derivatives **4**.

The Mannich reaction with aromatic aldehydes and cyclic amines was performed efficiently on 2-naphthol, by using SiO_2_-supported copper triflate under solvent-free conditions, without an additional co-catalyst or additive (Scheme 3) [17].

**Scheme 4:**
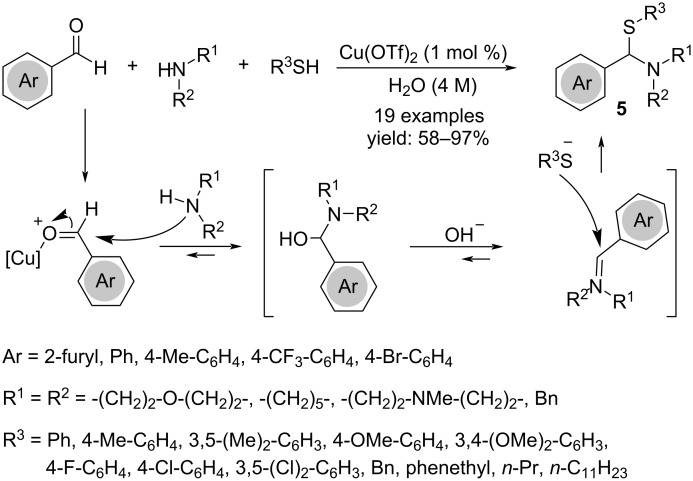
Synthesis of thioaminals **5**.

The treatment of stoichiometric amounts of arylaldehydes, secondary aliphatic or aromatic amines and thiols in the presence of catalytic Cu(OTf)_2_ (1 mol %) in aqueous media was proven to be a sustainable procedure to access thioaminals **5**, avoiding high temperatures and/or hazardous reagents required by classical conditions (Scheme 4) [18].

**Scheme 5:**
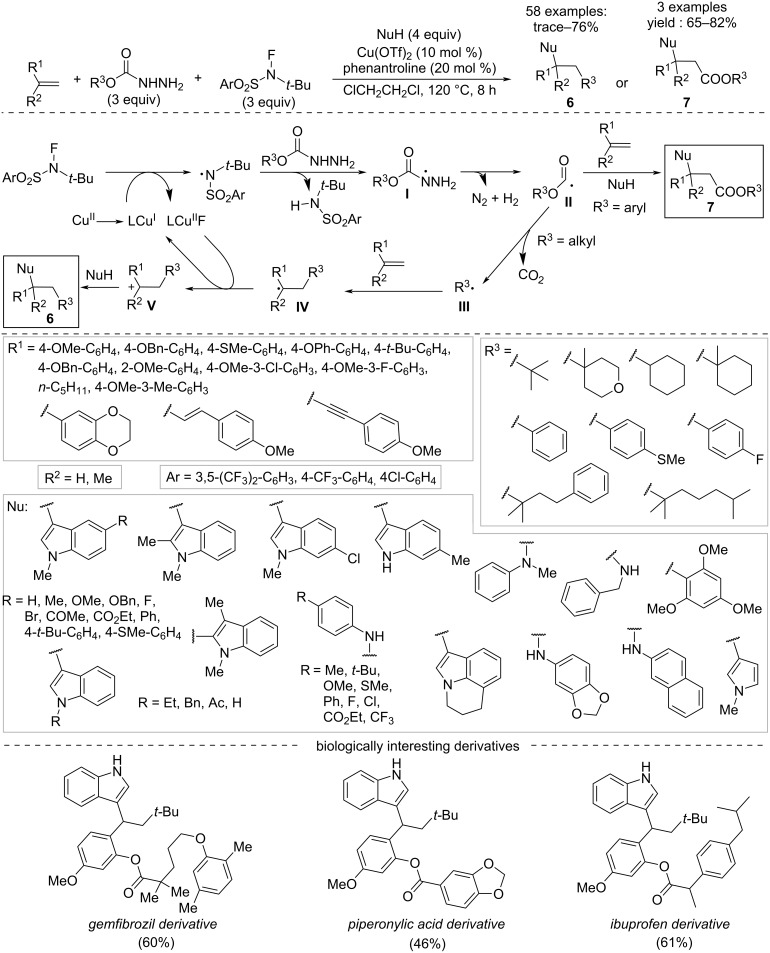
Synthesis of aryl- or amine-containing alkanes **6** and **7**.

The 1,2-difunctionalization of alkenes carried out with carbazates (*N*-aminocarbamates) and (hetero)arene nucleophiles or amines exploiting *N*-(*tert*-butyl)-*N*-fluoro-3,5-bis(trifluoromethyl)benzenesulfonamide (NFBS) as intermolecular hydrogen-atom-transfer reagent results in alkylarylation processes (Scheme 5) [19]. The reaction proceeds through an initial single-electron transfer from NFBS assisted by the active copper species, followed by intermolecular hydrogen-atom transfer from the carbazate. The nitrogen radical intermediate **I** thus formed is decomposed into the acyl or alkyl radical intermediates **II** and **III**, respectively. The latter interacts with the alkene generating an alkyl radical **IV** that converts to the cationic intermediate **V** by single-electron oxidation by the Cu(II) species. Finally, the attack of the nucleophile leads to the desired products **6**. Starting from aryl carbazates, intermediate **II**, adds directly to the alkene, then reacts with the nucleophile to afford product **7**.

**Scheme 6:**
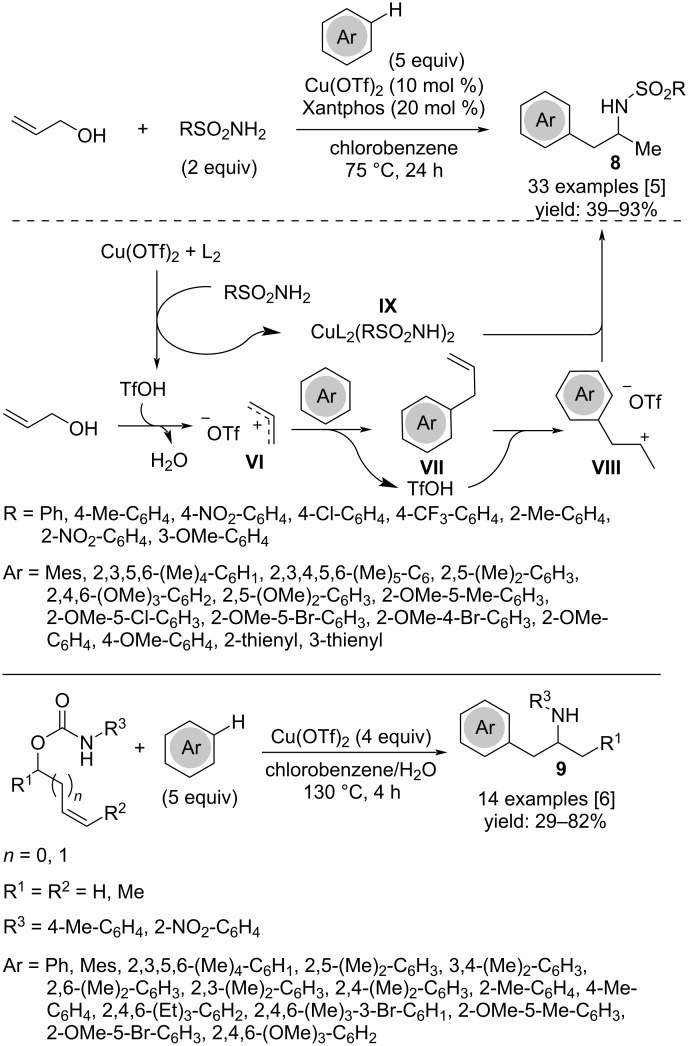
Synthesis of 1-aryl-2-sulfonamidopropanes **8**.

The regioselective 1,2-difunctionalization of allyl alcohol has been developed as a three-component cascade reaction using arenes and sulfonamides as nucleophiles to achieve arylation/hydroamination processes. The reaction involves a Friedel–Crafts alkylation of the arene followed by hydroamination (Scheme 6) [5]. The mechanism plausibly starts with the in situ formation of triflic acid from Cu(OTf)_2_ which leads to protonation of the oxygen atom of the alcohol with generation of the activated allyl alcohol. This latter gives the allyl carbenium ion **VI** through the loss of a molecule of water, then undergoes a Friedel–Crafts alkylation by attack of the aromatic partner. The outcome of the reaction proceeds through a Markovnikov protonation of the allylated arene **VII** by triflic acid, which generates the carbocation intermediate **VIII**. At this stage, the amido–copper complex **IX** selectively attacks the intermediate providing the 1-aryl-2-sulfonamidopropane **8**. This procedure is a valuable alternative to a similar approach for the synthesis of amphetamine derivatives **9** from allyl carbamates that requires excess of Cu(OTf)_2_ [6].

**Scheme 7:**
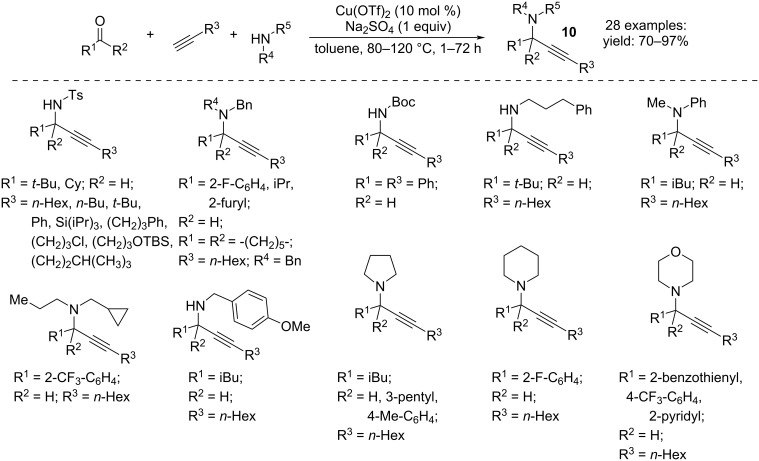
Synthesis of α-substituted propargylamines **10**.

Three-component coupling of amines, aldehydes or ketones, and terminal alkynes catalyzed by Cu(OTf)_2_ is a fruitful tool for the production of α-substituted propargylamines **10** (Scheme 7) [20]. The reaction involves the alkynylation of the corresponding imines formed in situ and provides higher yields than the two-step reactions. The addition of Na_2_SO_4_ facilitates the formation of the products, while MgSO_4_ or molecular sieves were found to be irrelevant to the reaction rate. It should be noted that this three-component process is promoted by the specific combination of Cu(II) with the triflate counteranion.

**Scheme 8:**
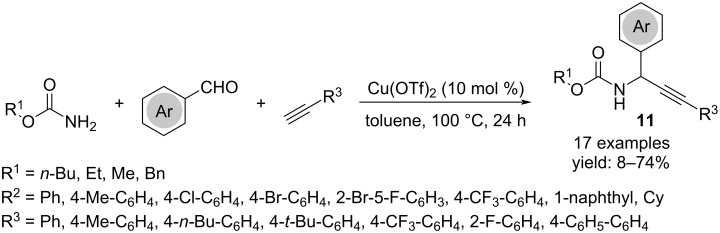
Synthesis of *N*-propargylcarbamates **11**.

The use of a carbamate among the substrates instead of the amine allowed the synthesis of propargylcarbamates **11**. This reaction, effective only for the aromatic aldehydes, did not require other co-catalysts or ligands (Scheme 8) [21].

**Scheme 9:**
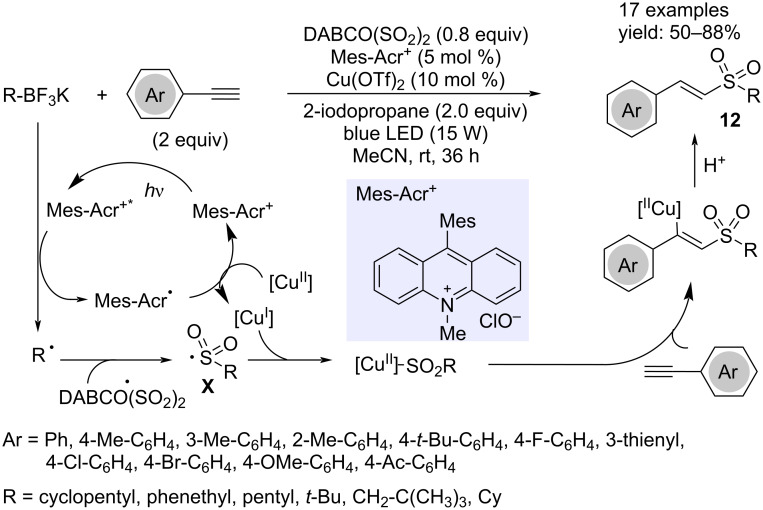
Synthesis of (*E*)-vinyl sulfones **12**.

Three-component reactions of alkynes, alkyltrifluoroborates and sulfur dioxide afforded vinyl sulfones with excellent regio- and stereoselectivity (Scheme 9) [22]. The authors used DABCO(SO_2_)_2_ to generate sulfur dioxide, and visible light irradiation and the mandatory presence of a photocatalyst for this transformation suggested a radical mechanism. The inhibition of the reaction in the presence of TEMPO confirmed this hypothesis. The copper catalyst assisted in the addition step of the alkylsulfonyl radical **X** to the alkyne. The presence of 2-iodopropane as additive improved the yields. The role was unclear, but it might facilitate the conversion of the alkyltrifluoroborate into its corresponding alkyl radical.

**Scheme 10:**
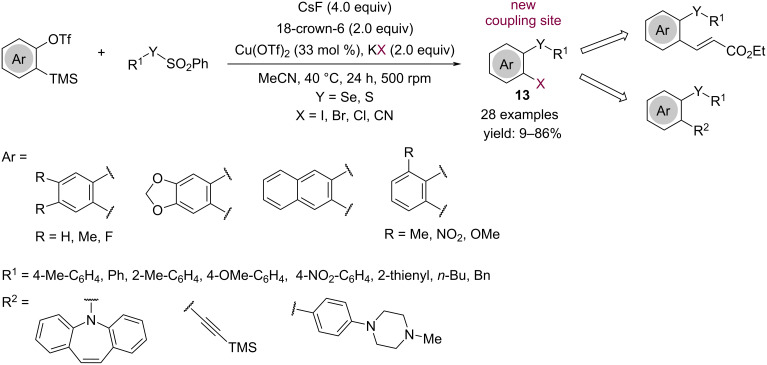
Synthesis of *o*-halo-substituted aryl chalcogenides **13**.

*o*-Halo-substituted aryl selenides and sulfides **13** can be achieved by a three-component coupling reaction performed with an aryne precursor, potassium halides and electrophilic chalcogen species as reactants, in the presence of Cu(OTf)_2_ (Scheme 10) [23]. Under these conditions the reaction between aryl thiosulfonates with arynes to give sulfones is competitive. Cu(OTf)_2_ is essential to remove the sulfinate anions in the reaction medium, avoiding side reactions arising from their attack to the electrophilic arynes. The so-obtained products are susceptible of Pd-catalyzed cross-coupling reactions, allowing the formation of C–C and C–N bonds in the *o*-position of the aryl chalcogen compounds.

**Scheme 11:**
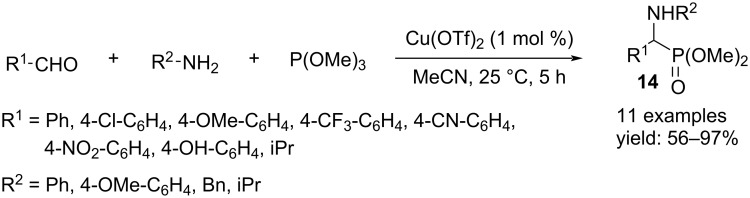
Synthesis of α-aminophosphonates **14**.

α-Aminophosphonates **14** were the result of a one-pot condensation of an aldehyde, a primary amine and phosphite P(OMe)_3_ with copper triflate acting as Lewis acid. Electron-poor and electron-rich aromatic aldehydes gave good results, whereas aliphatic aldehydes gave moderate yields (Scheme 11) [24].

**Scheme 12:**
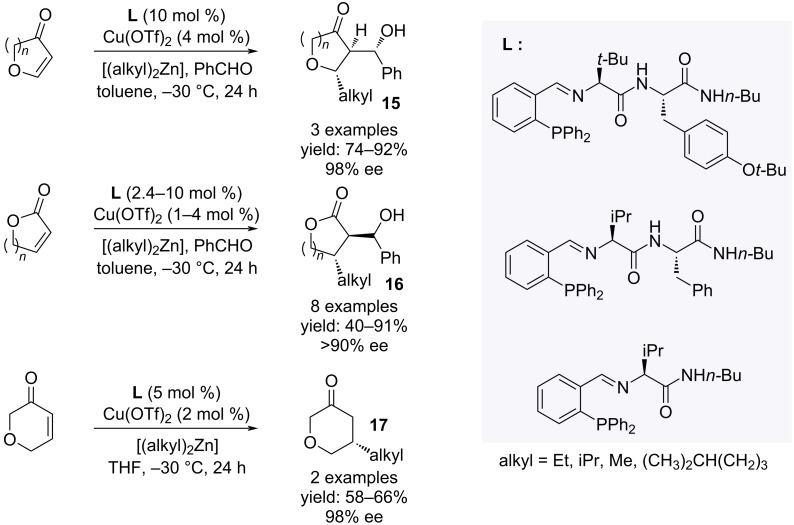
Synthesis of unsaturated furanones and pyranones **15**–**17**.

The asymmetric conjugate addition of dialkylzinc and benzaldehyde to unsaturated carbonyls under copper catalysis in the presence of optically pure phosphanes was realized with high diastereo- and enantioselectivities (Scheme 12) [25].

#### Providing cyclic compounds

**Scheme 13:**
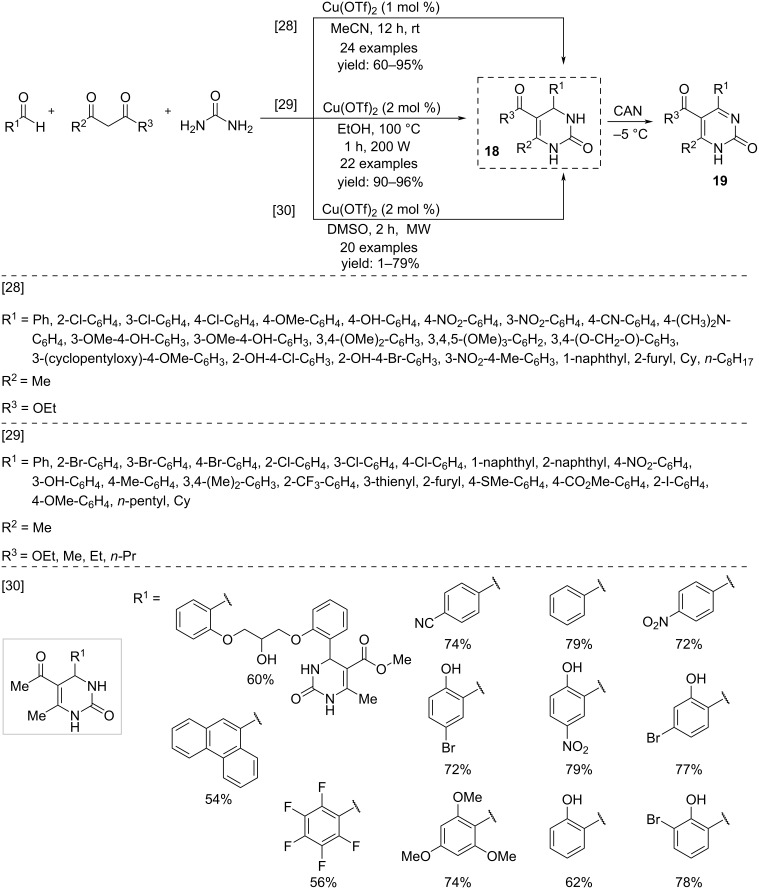
Synthesis of substituted dihydropyrimidines **18**.

For more than a century, Biginelli's reaction has been known as an effective tool for the construction of dihydropyrimidines through a three-component process by condensation in an acidic medium of an aldehyde, urea and a 1,3-dicarbonyl compound [26–27]. In these reactions, the use of catalytic Cu(OTf)_2_ proved to be an excellent triflate surrogate, also revealing a remarkable reuse activity. The first example of a Biginelli reaction carried out with Cu(OTf)_2_ catalysis was reported by Sudalai and co-workers in 2003 (Scheme 13) [28]. Working in acetonitrile at room temperature, very high yields were obtained with recycling of the catalyst with negligible loss of activity. The reaction is successful also by operating it in ethanol as a solvent under microwave irradiation [29]. More recently, the Biginelli reaction was carried out starting from salicylaldehyde providing hydroxyphenyl-substituted dihydropyrimidines **18** [30]. Subsequently, the regioselective oxidation of the dihydropyrimidine ring in the presence of CAN allowed the formation of new pyrimidinone derivatives **19**.

**Scheme 14:**
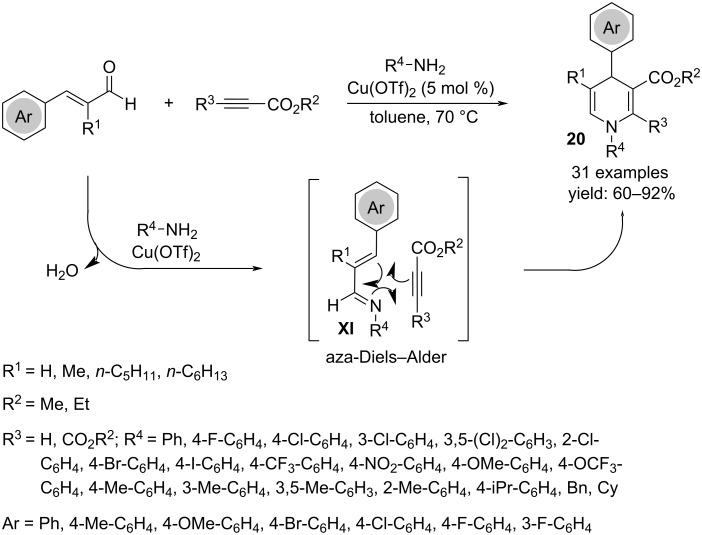
Regioselective synthesis of 1,4-dihydropyridines **20**.

The efficacy of Cu(OTf)_2_ as a catalyst in three-component processes was also demonstrated in three-component reactions involving alkynes, amines and α,β-unsaturated aldehydes to obtain 1,4-dihydropyridines **20** (Scheme 14) [31]. By using terminal alkynes, 2,6-unsubstituted products were achieved. Concerning the mechanism, it is plausible to assume as the key step for ring formation an aza-Diels–Alder reaction between the alkyne and the imine generated by dehydration between the aldehyde and aniline. The catalyst promotes the formation of the imine **XI**, while the high regioselectivity is ascribable to the favored orientation between the electron-rich nitrogen of the diene and the electron-poor carbon of the alkyne.

**Scheme 15:**
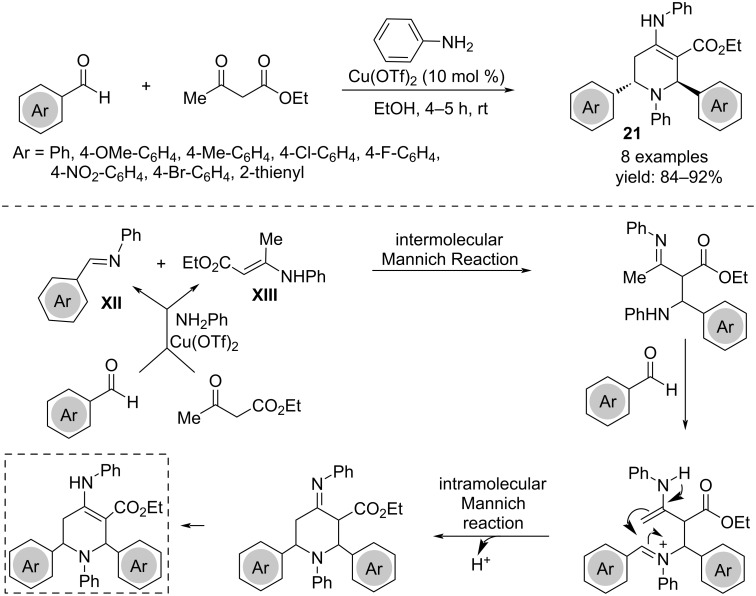
Synthesis of tetrahydropyridines **21**.

A different one-pot procedure affording tetrahydropyridines was developed employing two molecules of aromatic aldehydes, ethyl acetoacetate and two molecules of aniline. The copper triflate catalyst acts in the initial formation of imine **XII** and enamine **XIII**, reacting each other in a mechanism that involved two Mannich-type reactions (Scheme 15) [32].

**Scheme 16:**
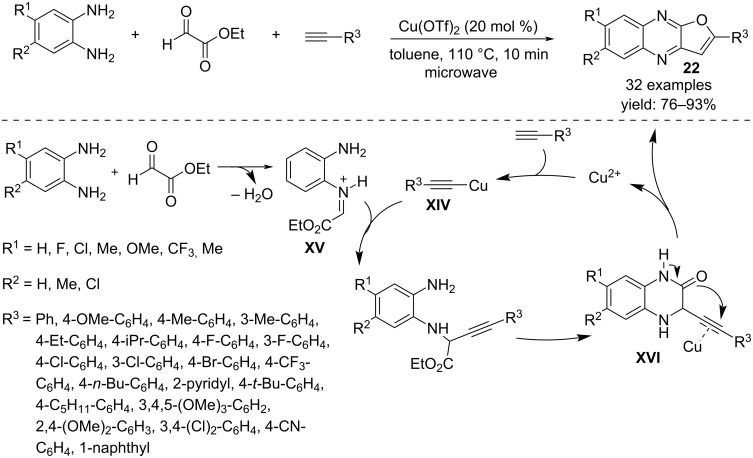
Synthesis of furoquinoxalines **22**.

Activation of terminal alkynes with Cu(OTf)_2_ is the key step for the preparation of furoquinoxalines **22** from *o*-phenylenediamine and ethyl glyoxylate (Scheme 16) [33]. The reaction, which occurs with formation of C–C, C–N and C–O bonds, involves a nucleophilic addition of the activated alkyne **XIV** to the in situ-generated iminium ion **XV**, followed by cyclization to form a quinoxalin-2-one intermediate **XVI**. A subsequent 5-*endo*-*dig* cyclization involving the triple bond furnishes the furo-ring and the final oxidation affords the tricyclic product **22**.

**Scheme 17:**
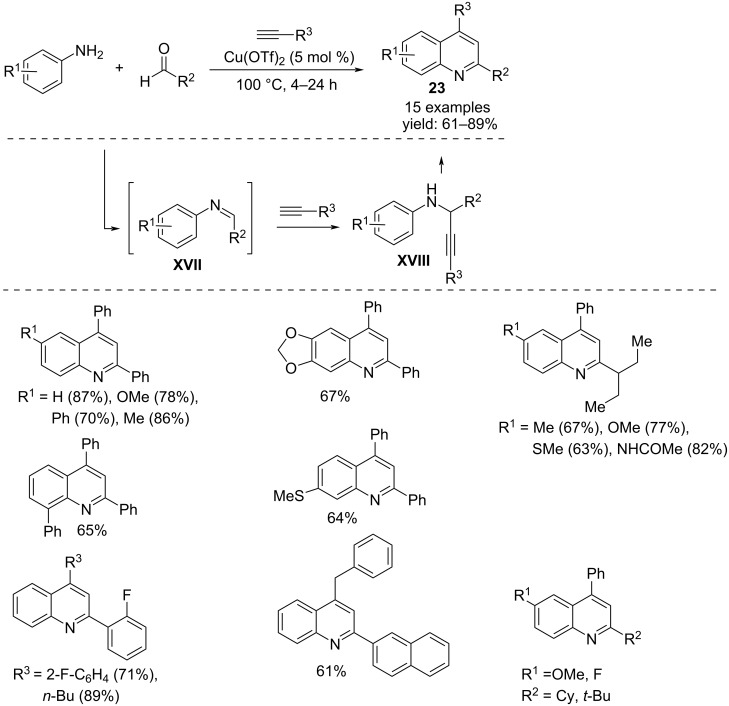
Synthesis of 2,4-substituted quinolines **23**.

Substituted quinolines **23** were obtained in a convenient solvent-free multicomponent reaction starting from electron-rich or electron-poor anilines, alkyl or arylaldehydes and terminal alkynes, performing the coupling with copper triflate as catalyst, without ligand, co-catalyst or other additives. The reaction involved the formation of the imine **XVII** followed by alkynylation to propargylamine **XVIII**, cyclization, and oxidation to quinoline **23** (Scheme 17) [34].

**Scheme 18:**
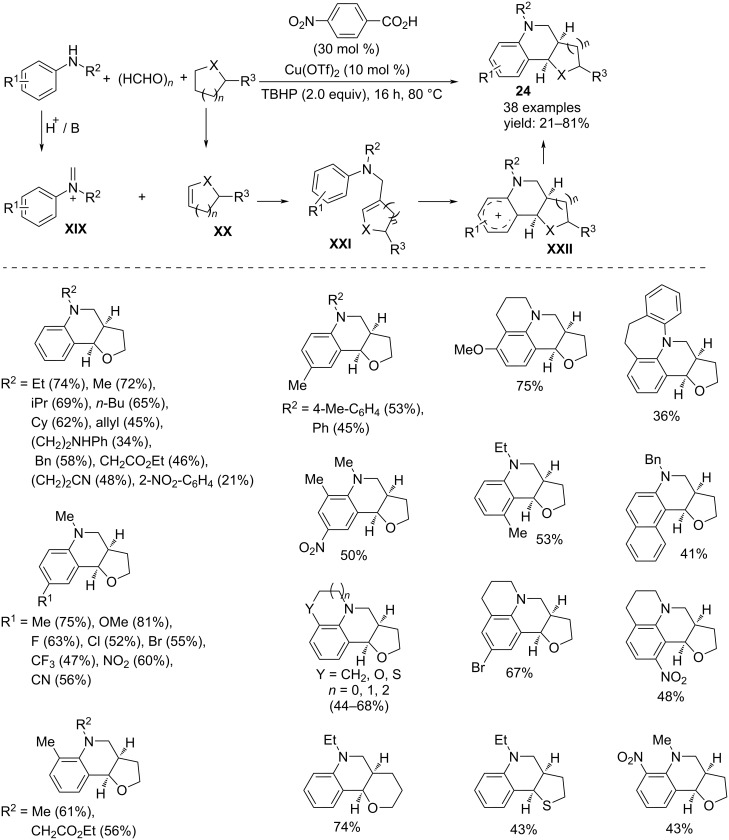
Synthesis of cyclic ether-fused tetrahydroquinolines **24**.

Three component oxidative annulation to obtain cyclic ether-fused tetrahydroquinolines **24** has been reported starting from secondary anilines, cyclic ethers and paraformaldehyde (Scheme 18) [35]. In addition to Cu(OTf)_2_ as a catalyst, the most effective reaction conditions required a substoichiometric amount of *p*-nitrobenzoic acid as an additive. Some control experiments support a mechanism whose key intermediates are the formation of the iminium ion **XIX**, originated from aniline with formaldehyde which serves as the C1 building block, and the generation of the cyclic α,β-unsaturated ethers **XX** by Cu(OTf)_2_-catalyzed dehydrogenation of the corresponding saturated compounds. Subsequent nucleophilic addition of the cyclic vinyl ether to the iminium salt generates an intermediate **XXI** susceptible of intramolecular electrophilic attack to give a tricyclic structure **XXII**. The final deprotonation provides the desired product **24**.

**Scheme 19:**
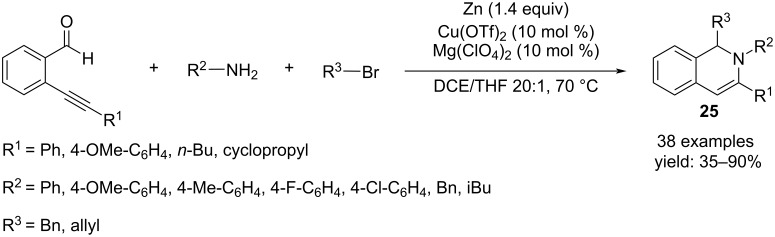
Practical route for 1,2-dihydroisoquinolines **25**.

The multicomponent reaction was also fruitful to obtain 1,2-dihydroisoquinolines **25** starting from 2-alkynylbenzaldehydes, primary amines and allylic or benzyl bromide, in the presence of zinc and using the combination of Mg(ClO_4_)_2_/Cu(OTf)_2_ as catalyst. The use of a mixture THF/DCE 1:20 as solvent was mandatory, because THF was crucial for the formation of the organozinc reagent (Scheme 19) [36].

**Scheme 20:**
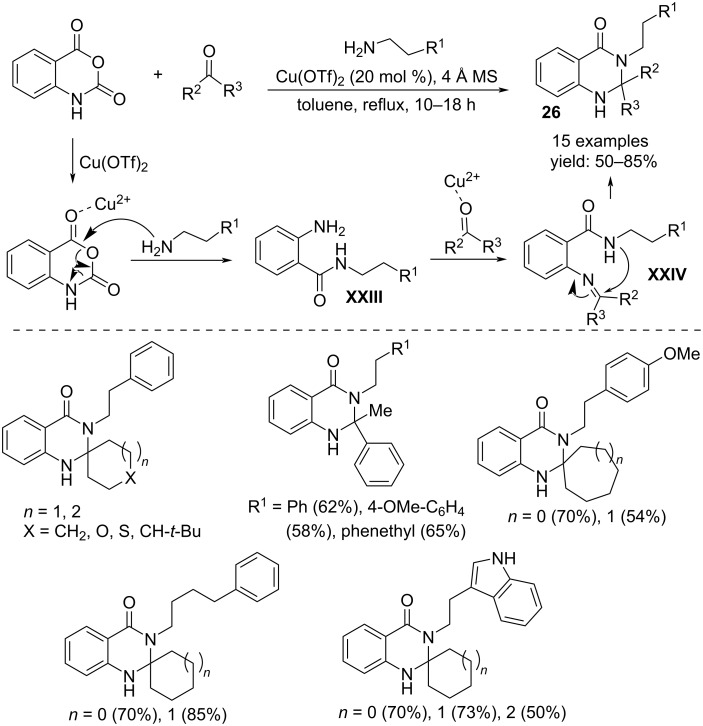
Synthesis of 2,3-dihydroquinazolin-4(1*H*)-one derivatives **26**.

Spiro-2,3-dihydroquinazolinones **26** were formed exploiting a one-pot multicomponent reaction, using isatoic anhydride, ketones and primary amines. The isolation of the amide intermediate **XXIII** obtained by the copper-catalyzed reaction between the anhydride and the amine suggested the subsequent reaction with the ketone to give an imine intermediate **XXIV**. This latter can undergo intramolecular nucleophilic attack affording the quinazolinone derivative **26** (Scheme 20) [37].

**Scheme 21:**
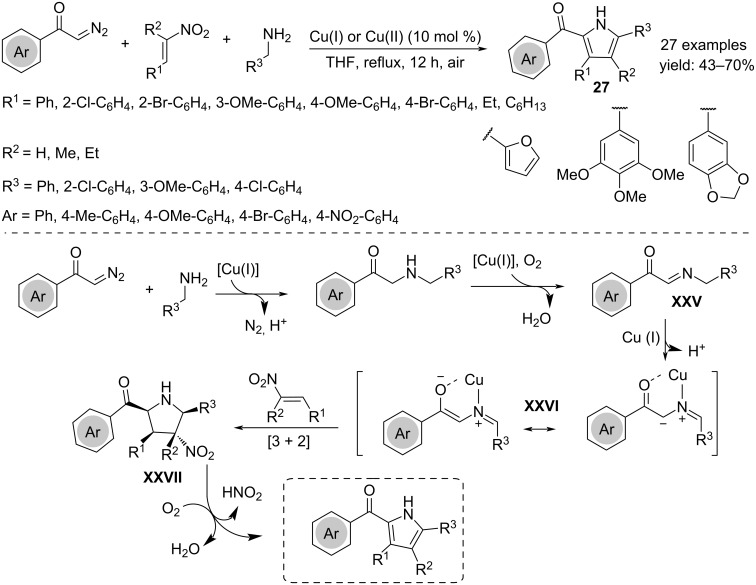
Synthesis of polysubstituted pyrroles **27**.

Polysubstituted pyrroles **27** were obtained in a cascade process by using α-diazoketones, nitroalkenes and primary amines, in the presence of air as oxidant. The mechanism involved the formation of α-ketocarbene **XXVI** from α-diazoketone, able to react with the amine affording imine **XXV** after copper-catalyzed oxidative dehydrogenation. The subsequent [3 + 2] cycloaddition reaction with the nitroalkene produces the pyrrolidine **XXVII**, which then aromatizes by extrusion of HNO_2_ (Scheme 21) [38].

**Scheme 22:**
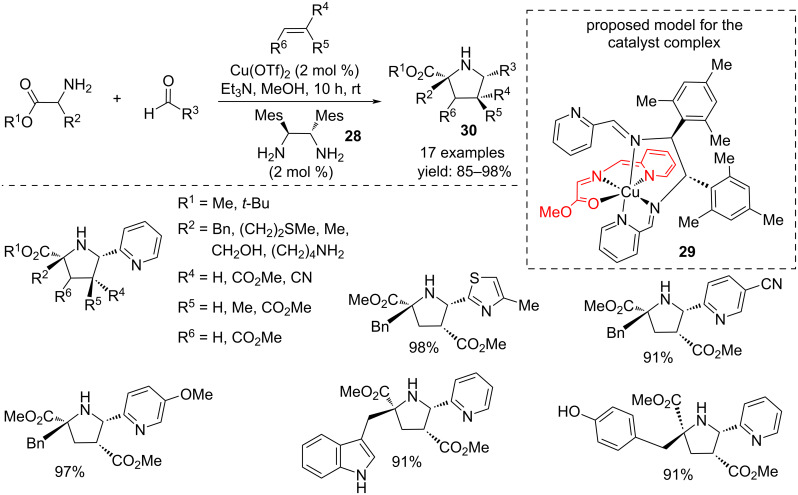
Enantioselective synthesis of polysubstituted pyrrolidines **30** directed by the copper complex **29**.

Substituted pyrrolidines **30** were achieved in an enantioselective form starting from amino acid esters, electron-poor olefins and 4-substituted-2-picolinaldehydes or 4-methylthiazole-2-carboxaldehyde as chelating agent, in the presence of copper triflate and the chiral diamine ligand **28**. The stereoselectivity was directed by the formation of a proposed catalyst complex **29** involving two molecules of Schiff base (Scheme 22) [39].

**Scheme 23:**
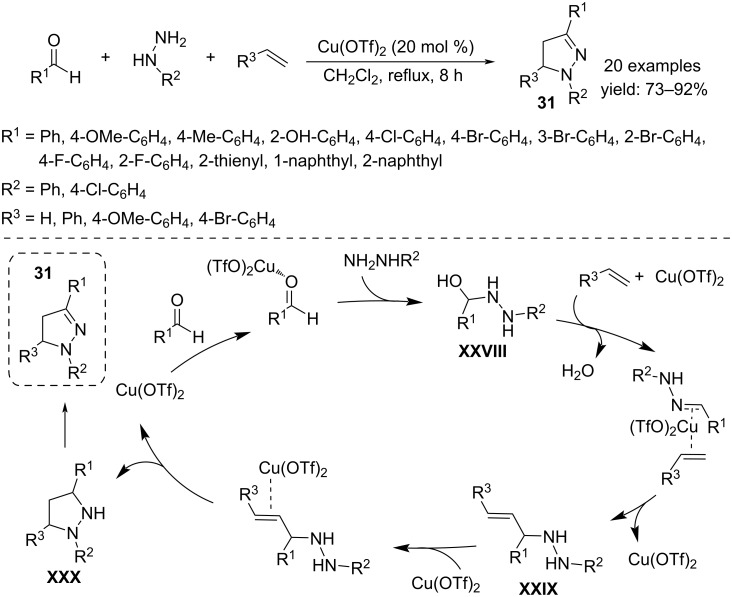
Synthesis of 4,5-dihydropyrazoles **31**.

The three-component annulation of aldehydes, hydrazines and alkenes with Cu(OTf)_2_ (20 mol %) in CH_2_Cl_2_ at reflux is a useful tool to access substituted 4,5-dihydropyrazoles **31** (Scheme 23) [40]. The products reasonably result from a Mannich/cyclization/oxidative transformation of the substrates in which Cu(OTf)_2_ is involved in more steps. The reaction begins with a nucleophilic attack of hydrazine on the aldehyde, activated by the copper salt, to give the corresponding hydrazone **XXVIII**. Subsequently, the formation of a Mannich-type intermediate **XXIX** was hypothesized by interaction between the hydrazone and the alkene mediated by Cu(OTf)_2_ coordination, which favors the approach of the reaction centers. It is again a metal coordination that activates the C–C double bond towards an intramolecular reaction to give the tetrahydropyrazole **XXX** via formation of a C–N bond. The final oxidation in air gives the 4,5-dihydropyrazole **31**.

**Scheme 24:**
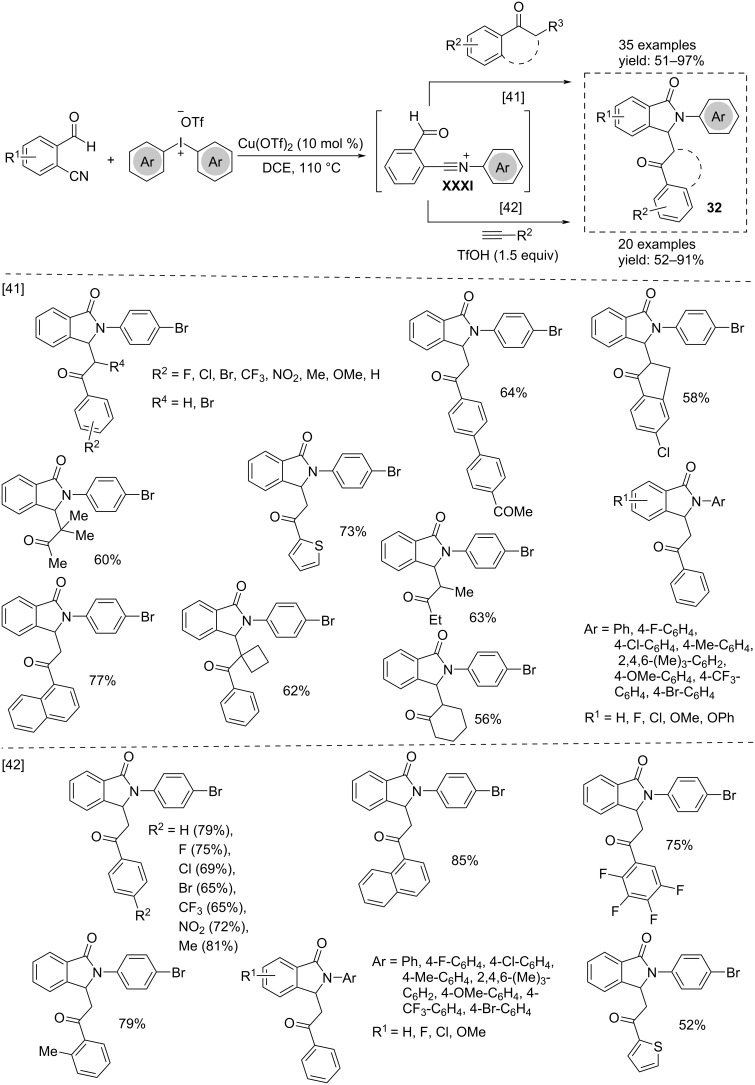
Synthesis of 2 arylisoindolinones **32**.

Cu(OTf)_2_ is also capable of promoting the three-component cascade cyclization of 2-formylbenzonitriles, alkyl aryl ketones, and diaryliodonium salts to afford 2-arylisoindolinones **32** (Scheme 24) [41]. It is conceivable that the reaction starts with the formation of an *N*-arylnitrilium cation **XXXI** that, after hydrolysis, reacts with an enol species activated by the copper catalyst, affording the final product. The same research group reported an extension of this study by starting from arylacetylenes instead of arylketones [42].

**Scheme 25:**
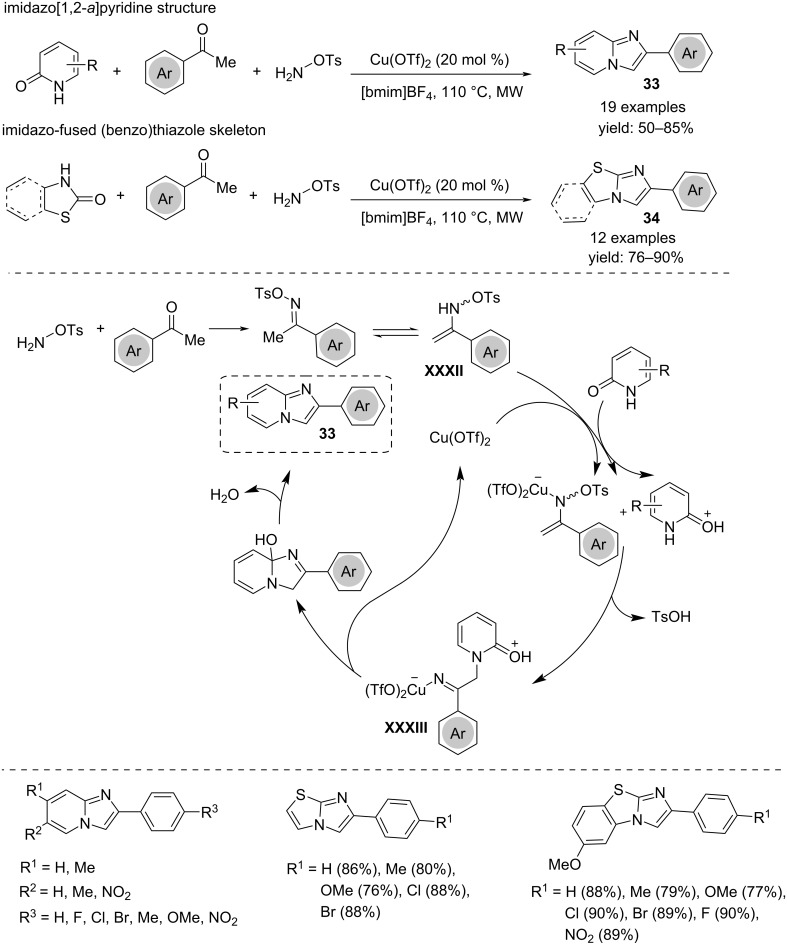
Synthesis of imidazo[1,2-*a*]pyridines **33**.

Imidazo[1,2-*a*]pyridine derivatives **33** can be achieved by Cu(OTf)_2_-catalyzed multicomponent reactions starting from different reagents. In a first approach proposed by Meshram and co-workers, pyridin-2-one, *O*-tosylhydroxylamine and acetophenone treated in an ionic liquid assembled through the cascade formation of three C–N bonds to give the imidazo[1,2-*a*]pyridine scaffold **33** (Scheme 25) [43]. The reaction is facilitated under microwave irradiation and can be extended to the preparation of an imidazo-fused (benzo)thiazole skeleton **34** starting from (benzo)thiazol-2-ones instead of pyridin-2-ones. Moreover, the Cu(OTf)_2_ in [bmim]BF_4_ can be recovered and reused for multiple processes. The key step of the mechanism is the attack of the protonated pyridin-2-one to the copper-complex of the enamine **XXXII** resulting from the reaction between acetophenone and *O*-tosylhydroxylamine, which occurs with elimination of TsOH. The so-obtained imino–copper complex **XXXIII** gives rise to an intramolecular C–N bond formation releasing Cu(OTf)_2_. The final bicyclic product **33** arises from isomerization and water elimination.

**Scheme 26:**
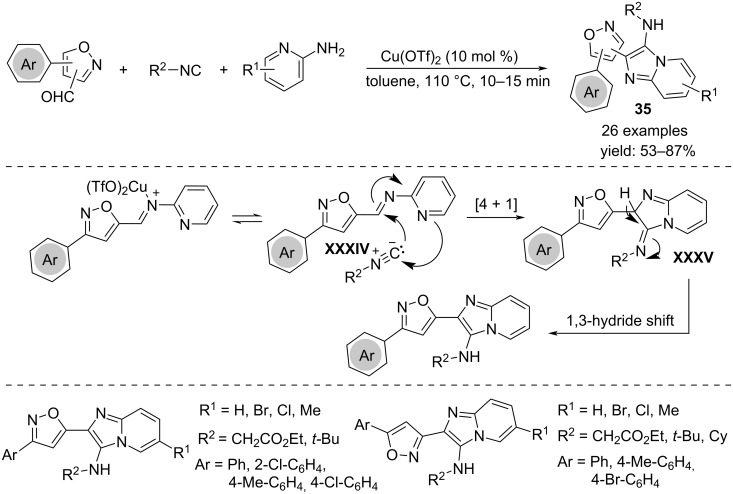
Synthesis of isoxazole-linked imidazo[1,2-*a*]azines **35**.

Recently, Singh's research group developed a cascade process to access imidazo[1,2-*a*]pyridines-linked isoxazoles **35**. Isoxazole carbaldehydes treated with 2-aminopyridines and isonitriles in the presence of catalytic amounts of Cu(OTf)_2_ lead to the formation of the products through formation of one C–C bond and three C–N bonds (Scheme 26) [44]. The same procedure allows a more general scope, giving access to imidazo[1,2-*a*]pyrimidine, imidazo[1,2-*a*]pyrazine and imidazo[2,1-*b*]thiazole derivatives. From the mechanistic point of view, it is expected that the reaction proceeds via formation of an imine **XXXIV** between isoxazole carbaldehyde, activated by the copper salt, and 2-aminoazine, which in turn undergoes a non-concerted [4 + 1] cycloaddition involving isonitrile to give the imidazole ring of intermediate **XXXV**. Finally, the final product **35** is yielded via a 1,3-hydride shift.

**Scheme 27:**
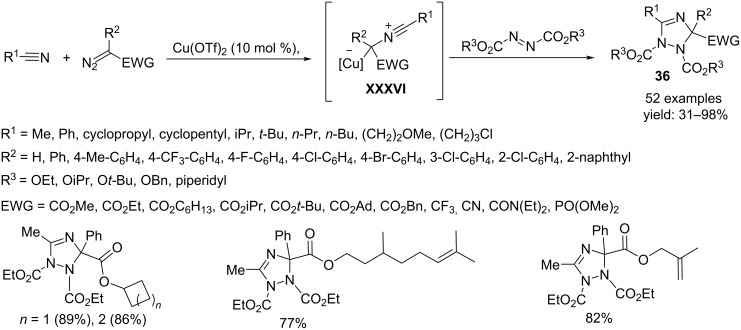
Synthesis of 2,3-dihydro-1,2,4-triazoles **36**.

The reaction between diazo derivatives, nitriles, and azodicarboxylates catalyzed by Cu(OTf)_2_ is an efficient synthetic method to obtain 2,3-dihydro-1,2,4-triazole derivatives **36** (Scheme 27) [45]. The reaction proceeds via a [3 + 2] cycloaddition reaction between azodicarboxylates and nitrile ylides **XXXVI** as 1,3-dipoles. The latter are generated from diazoalkanes under the coordination of the copper catalyst to form a carbenoid species that undergoes nucleophilic attack of the nitriles. This transformation has demonstrated high tolerance to functional groups and runs, under mild conditions, with electron-poor diazo derivatives such as 2-diazoacetate, 2-diazoacetonitrile, 2-diazo-1,1,1-trifluoromethane, diazoamide, and diazophosphonate.

**Scheme 28:**
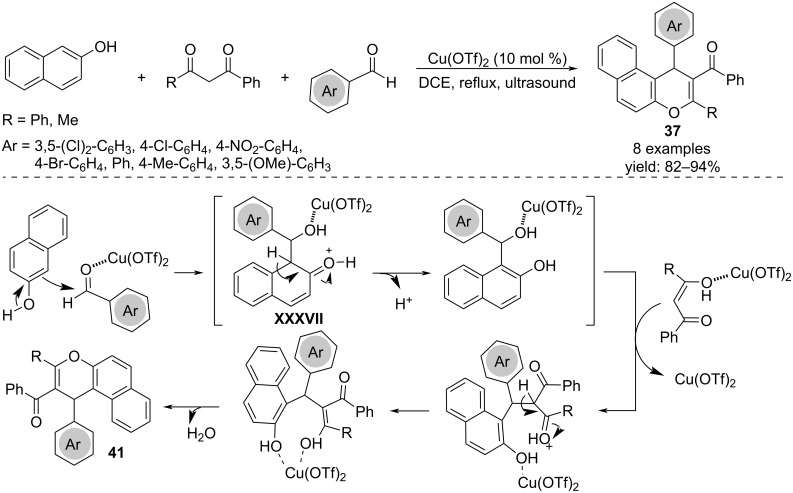
Synthesis of naphthopyrans **37**.

Condensation of 2-naphthol, aromatic aldehydes and acyclic 1,3-dicarbonyl compounds catalyzed by copper triflate under ultrasound irradiation allowed the one-pot formation of 1*H*-benzo[*f*]chromen-2-yl(phenyl)methanones (naphthopyranes) **37**. The comparison with conventional method showed better yields and shorter reaction times. The suggested reaction mechanism showed the formation of an *ortho*-quinone methide intermediate **XXXVII** formed through nucleophilic attack of the 2-naphthol to the aldehyde followed by reaction with 1,3-dicarbonyl compound coordinated by the copper. The subsequent intramolecular nucleophilic attack of the oxygen to the enol and water elimination resulted in the final product **37** (Scheme 28) [46].

**Scheme 29:**
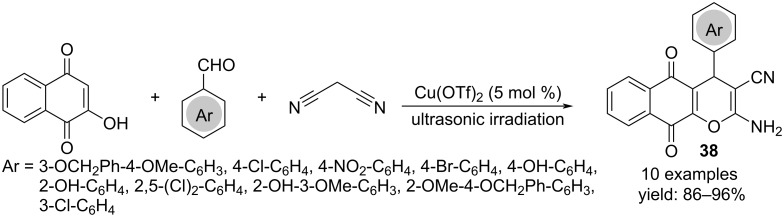
Synthesis of benzo[*g*]chromene derivatives **38**.

Analogously, benzo[*g*]chromene derivatives **38** were achieved starting from 2-hydroxynaphthalene-1,4-dione, aromatic aldehydes and malononitrile with copper triflate as catalyst and ultrasonic irradiation (Scheme 29) [47].

**Scheme 30:**
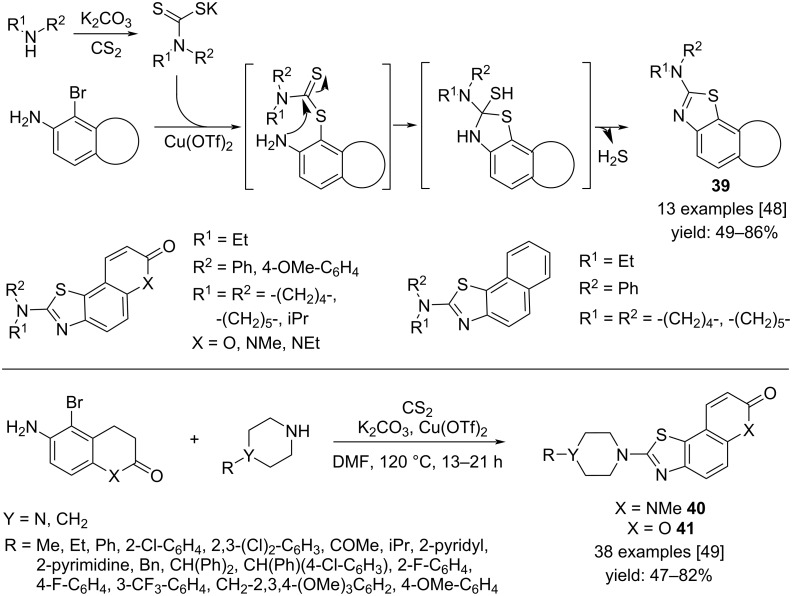
Synthesis of naphthalene annulated 2-aminothiazoles **39**, piperazinyl-thiazoloquinolines **40** and thiazolocoumarins **41**.

Naphthalene-annulated 2-aminothiazoles **39** were prepared exploiting the multicomponent reaction by using aminonaphthalenes, CS_2_ and secondary amines. The mechanism involved the Ullmann-type coupling of the bromo(amino)naphthalene with the dithiocarbamate salt followed by intramolecular nucleophilic attack of the naphthalene amino group to the C=S bond. The subsequent elimination of H_2_S afforded the final product (Scheme 30) [48].

Analogously, piperazinylthiazoloquinolines **40** and thiazolocoumarins **41** were obtained using piperazine or piperidine, CS_2_ and substituted quinolines or coumarins [49].

**Scheme 31:**
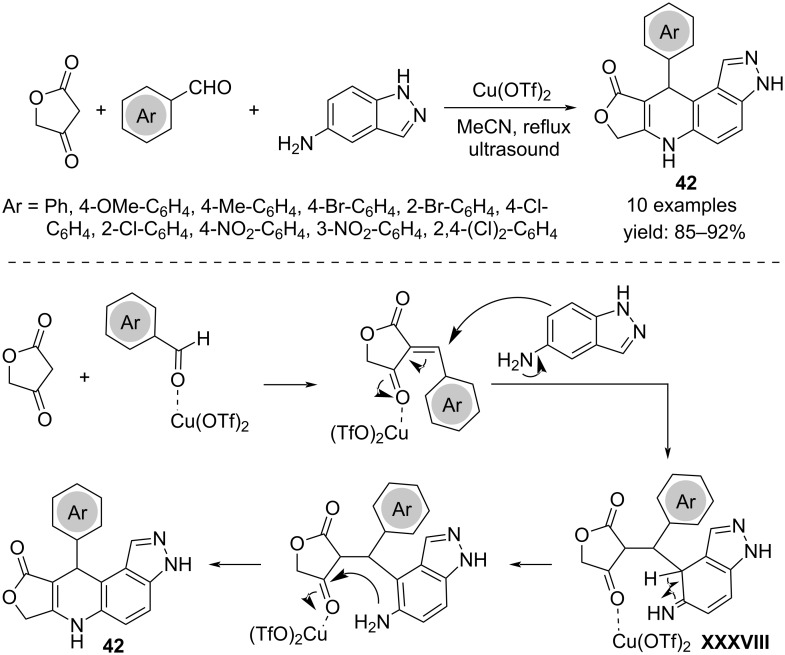
Synthesis of furo[3,4-*b*]pyrazolo[4,3-*f*]quinolinones **42**.

The synthesis of furo[3,4-*b*]pyrazolo[4,3-*f*]quinolinones **42** was achieved via the one-pot reaction of tetronic acid, 5-aminoindazole and arylaldehydes under copper catalysis and ultrasonic irradiation, in acetonitrile as solvent. The mechanism involves a Knoevenagel condensation between the tetronic acid and the arylaldehyde as first step, followed by a Michael-type addition of 5-aminoindazole to afford the first coupling product **XXXVIII**. The subsequent intramolecular amination and dehydration then leads to the final product (Scheme 31) [50].

**Scheme 32:**
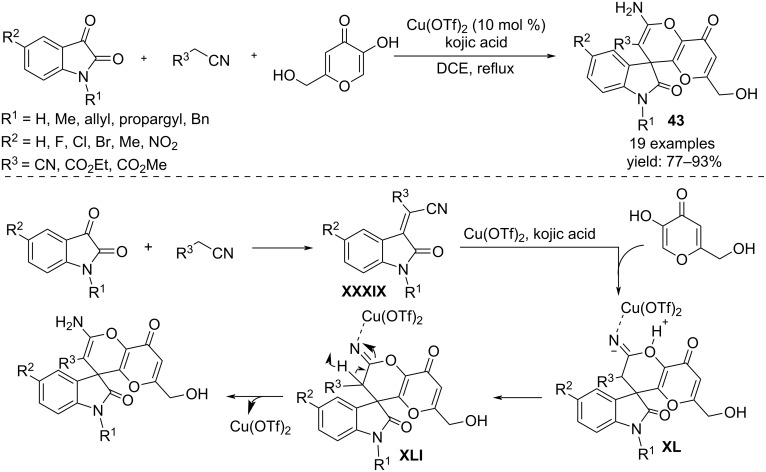
Synthesis of spiroindoline-3,4’-pyrano[3,2-*b*]pyran-4-ones **43**.

Polycyclic spiroindoline-3,4’-pyrano[3,2-*b*]pyran-4-ones **43** were synthesized exploiting the three-component reaction of isatin, 5-hydroxy-2-(hydroxymethyl)-4*H*-pyran-4-one (i.e. kojic acid) and malononitriles or cyanoacetates (Scheme 32) [51]. Compared to other Lewis acids, Cu(OTf)_2_ proved to be the best. Mechanistically, the process begins with a Knoevenagel-type condensation between isatin and the cyano derivative, yielding a 3-alkylidene-substituted oxindole **XXXIX** that, after coordination with Cu(OTf)_2_, is able to react with the enolic form of kojic acid to generate the C-alkylated intermediate **XL**. Subsequent intramolecular nucleophilic attack of the enolic hydroxy group to the copper-activated cyano group, results in a spiro-cyclized intermediate **XLI** that affords the final product by deprotonation and loss of the copper species.

### Four-component reactions

**Scheme 33:**
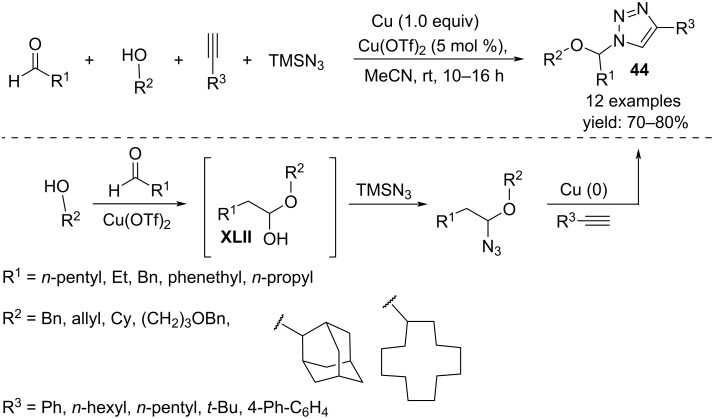
Synthesis of *N*-(α-alkoxy)alkyl-1,2,3-triazoles **44**.

Two different four-component procedures catalyzed by Cu(OTf)_2_ are reported in the literature, both to access 1,2,3-triazole derivatives. The first one is a cascade reaction for the preparation of α-alkoxy-*N*-alkyltriazoles **44** that was developed starting from aliphatic aldehydes, alcohols, TMSN_3_ as azide source and alkynes (Scheme 33) [52]. The reaction occurs under mild conditions in acetonitrile at room temperature but is inhibited when using aromatic aldehydes and phenols. The mechanism involves the reaction of the azide with the hemiacetal **XLII** generated in situ from the aldehydes and alcohols, followed by coupling with the alkynes to form the triazole ring. Both, copper triflate and copper metal are essential for the success of the reaction.

**Scheme 34:**
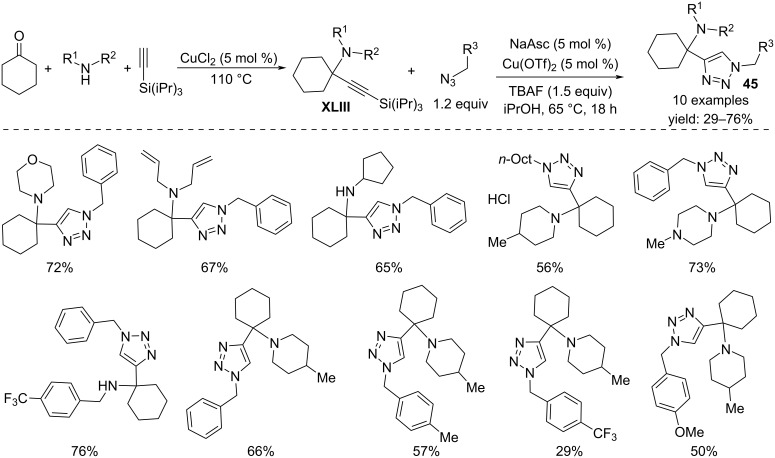
Synthesis of 4-(α-tetrasubstituted)alkyl-1,2,3-triazoles **45**.

On the other hand, 4-(α-tetrasubstituted)alkyl-1,2,3-triazoles **45** can be obtained by a two-step reaction of cyclohexanone, amines, silylacetylene, and aryl or alkyl azides in the presence of copper(II) catalysts (Scheme 34) [53]. In a first step, there is the formation of a propargylamine derivative **XLIII**, followed by silyl deprotection and azide cycloaddition resulting in the triazole product. The presence of Cu(OTf)_2_ as the catalyst, sodium ascorbate as a mild reductant and TBAF to deprotect the alkyne moiety are crucial in the cycloaddition step.

## Conclusion

In this review the developments on the multicomponent synthesis of acyclic and heteropolycyclic systems under copper(II) triflate catalysis are reported. Using alkenes and alkynes as substrates, various types of reactions were considered, including hydroamination, condensation, cross-coupling, C–H functionalization, cycloaddition, aza-Diels–Alder, also in regio- and stereoselective processes. The interest for these strategies arises from the cost-effectiveness as one-pot processes, the ease of application and the great efficiency when directed to the synthesis of biologically active compounds.

## Data Availability

Data sharing is not applicable as no new data was generated or analyzed in this study.

## References

[1] Aneeja T, Neetha M, Afsina C M A, Anilkumar G (2020). RSC Adv.

[2] Chemler S R (2015). Beilstein J Org Chem.

[3] Tschan M J-L, Thomas C M, Strub H, Carpentier J-F (2009). Adv Synth Catal.

[4] Rosenfeld D C, Shekhar S, Takemiya A, Utsunomiya M, Hartwig J F (2006). Org Lett.

[5] Loro C, Papis M, Foschi F, Broggini G, Poli G, Oble J (2023). J Org Chem.

[6] Loro C, Oble J, Foschi F, Papis M, Beccalli E M, Giofrè S, Poli G, Broggini G (2022). Org Chem Front.

[7] Taylor J G, Whittall N, Hii K K (Mimi) (2005). Chem Commun.

[8] Hertweck C (2000). J Prakt Chem.

[9] Chemler S R (2011). J Organomet Chem.

[10] Rao W, Kothandaraman P, Koh C B, Chan P W H (2010). Adv Synth Catal.

[11] Ton T M U, Himawan F, Chang J W W, Chan P W H (2012). Chem – Eur J.

[12] Ghorai M K, Ghosh K, Das K (2006). Tetrahedron Lett.

[13] Asao N, Kasahara T, Yamamoto Y (2003). Angew Chem, Int Ed.

[14] Motornov V A, Tabolin A A, Nelyubina Y V, Nenajdenko V G, Ioffe S L (2021). Org Biomol Chem.

[15] Paraskar A S, Sudalai A (2006). Tetrahedron Lett.

[16] Muraki T, Fujita K-i, Terakado D (2006). Synlett.

[17] Dindulkar S D, Puranik V G, Jeong Y T (2012). Tetrahedron Lett.

[18] Cavaca L A S, Gomes R F A, Afonso C A M (2022). Molecules.

[19] Cheng C, Chen D, Li Y, Xiang J-N, Li J-H (2023). Org Chem Front.

[20] Meyet C E, Pierce C J, Larsen C H (2012). Org Lett.

[21] Dou X-Y, Shuai Q, He L-N, Li C-J (2010). Adv Synth Catal.

[22] Liu T, Ding Y, Fan X, Wu J (2018). Org Chem Front.

[23] Mindner J, Rombach S, Werz D B (2024). Org Lett.

[24] Paraskar A S, Sudalai A (2006). ARKIVOC.

[25] Brown M K, Degrado S J, Hoveyda A H (2005). Angew Chem, Int Ed.

[26] Biginelli P (1893). Gazz Chim Ital.

[27] Tron G C, Minassi A, Appendino G (2011). Eur J Org Chem.

[28] Paraskar A S, Dewkar G K, Sudalai A (2003). Tetrahedron Lett.

[29] Pasunooti K K, Chai H, Jensen C N, Gorityala B K, Wang S, Liu X-W (2011). Tetrahedron Lett.

[30] Huseynzada A E, Jelch C, Akhundzada H V N, Soudani S, Ben Nasr C, Israyilova A, Doria F, Hasanova U A, Khankishiyeva R F, Freccero M (2021). RSC Adv.

[31] Li S, Yang Q, Wang J (2016). Tetrahedron Lett.

[32] Patil K N, Mane R A, Jadhav S B, Mane M M, Helavi V B (2019). Chem Data Collect.

[33] Naresh G, Kant R, Narender T (2014). Org Lett.

[34] Meyet C E, Larsen C H (2014). J Org Chem.

[35] Duan P, Sun J, Zhu Z, Zhang M (2023). Org Biomol Chem.

[36] Gao K, Wu J (2007). J Org Chem.

[37] Zhu X, Kang S R, Xia L, Lee J, Basavegowda N, Lee Y R (2015). Mol Diversity.

[38] Hong D, Zhu Y, Li Y, Lin X, Lu P, Wang Y (2011). Org Lett.

[39] Chaulagain M R, Felten A E, Gilbert K, Aron Z D (2013). J Org Chem.

[40] Wu Q, Liu P, Pan Y-m, Xu Y-l, Wang H-s (2012). RSC Adv.

[41] Liu L, Bai S-H, Li Y, Ding X-D, Liu Q, Li J (2018). Adv Synth Catal.

[42] Li Y, Li Y, Fei H, Kong R, Yu Z, He L (2022). J Chem Res.

[43] Kumar G S, Ragini S P, Kumar A S, Meshram H M (2015). RSC Adv.

[44] Singh D, Sharma S, Thakur R K, Vaishali, Nain S, Jyoti, Malakar C C, Singh V (2024). Tetrahedron.

[45] Cai B-G, Li Q, Xuan J (2024). Green Synth Catal.

[46] Zeleke T Y, Turhan K, Turgut Z (2016). Am Chem Sci J.

[47] Perumal M, Sengodu P, Venkatesan S, Srinivasan R, Paramsivam M (2017). ChemistrySelect.

[48] Majumdar K C, Nirupam D, Ghosh D, Ponra S, Roy B (2012). Synthesis.

[49] Patel R V, Patel J K, Nile S H, Park S W (2013). Arch Pharm (Weinheim, Ger).

[50] Damavandi S, Sandaroos R, Mohammadi A (2013). Heterocycl Commun.

[51] Parthasarathy K, Praveen C, Balachandran C, Senthil kumar P, Ignacimuthu S, Perumal P T (2013). Bioorg Med Chem Lett.

[52] Yadav J S, Subba Reddy B V, Madhusudhan Reddy G, Rehana Anjum S (2009). Tetrahedron Lett.

[53] Palchak Z L, Nguyen P T, Larsen C H (2015). Beilstein J Org Chem.

